# Of the article: evaluation of the masticatory efficiency of children with stainless steel crowns: a pilot cross-sectional split-mouth study

**DOI:** 10.1186/s12903-024-04960-w

**Published:** 2024-10-01

**Authors:** Madhura Sen, Karuna Yarmunja Mahabala, Srikant Natarajan, Deveshi Gupta, Shrehya Shekhar, Ashwin Rao, Anupama Nayak P

**Affiliations:** 1 Department of Pediatric and Preventive Dentistry, Manipal College of Dental Sciences, Mangalore, Manipal Academy of Higher Education, Manipal, Karnataka India 576104; 2 Department of Oral Pathology, Manipal College of Dental Sciences, Mangalore, Manipal Academy of Higher Education, Manipal, Karnataka India 576104,

**Keywords:** Stainless steel crowns, Children, Masticatory efficiency, Chewing

## Abstract

**Background:**

Stainless steel crowns (SSCs) are commonly employed to restore the posterior teeth of children and over the years, they are available with improved anatomical shapes. This study was conducted to evaluate and assess the effect of the placement of SSCs on the masticatory efficiency of children.

**Methods:**

This pilot cross-sectional split-mouth study assessed masticatory efficiency in children aged 6–11 years. Fifteen participants, each with stainless-steel crowns placed unilaterally at least one month prior, were included. This study utilized two flavours of trident chewing gum (red and green) to measure masticatory efficiency. The child was asked to chew half a strip of red and green chewing gum placed one on top of the other using either the noncrown or crown side 15 times. Another set of chewing gum was given to the child to chew 20 times on the same side. The chewing exercise was repeated using teeth on the other side. Chewed gum samples were collected, photographed, and analysed via ImageJ software to determine the red and green areas. Masticatory efficiency was subsequently calculated with the help of a formula in which the red areas and green areas were calculated via ImageJ software. The data were analysed with paired t tests via SPSS (version 20.0).

**Results:**

When the mean values of the crown side were compared with those of the noncrown side following 15 chewing cycles, the chewing efficiency on the noncrown side was greater, with a difference of 0.303, whereas the chewing efficiency following 20 chewing cycles was greater on the crown side, with a difference of 0.814. However, both differences were statistically nonsignificant, with t values of -0.07 and 0.26, respectively, and p values of 0.94 and 0.8, respectively.

**Conclusion:**

The presence of SSCs on the molars of children did not affect masticatory efficiency.

## Background

Mastication, the initial stage of the digestive process, plays a critical role in breaking down ingested food, thereby facilitating optimal nutrient absorption and overall health. In children, effective mastication is not only vital for nutritional intake but also essential for the normal development of the maxilla and mandible. A reduction in masticatory efficiency can adversely affect physical development and overall quality of life [1, 2].

Occlusion refers to contact between the maxillary and mandibular dental arches and is crucial for effective mastication or chewing [3]. Occlusal interference or premature dental contact can result in uncoordinated masticatory movements, thereby decreasing masticatory efficiency [4]. Notably, occlusion in children is often underexplored in the pediatric restorative literature, even though restoring functional contact relationships is recommended after restorative treatments [5].

Stainless steel crowns (SSCs) are commonly employed in pediatric dentistry to restore occlusion in cases of extensive carious lesions and developmental defects. Compared with alternative restorative materials such as amalgam, glass ionomer cement (GIC), composites, or compomers, SSCs are routinely recommended for children with high caries risk or behavioral challenges, as SSCs provide a durable and reliable solution [6, 7].

The placement of stainless steel crowns in children results in short-term premature contact and a decrease in biting force, which is said to gradually return to normal [8]. Over the years, manufacturers have made several modifications to enhance their efficacy, including enhancements for a more precise anatomical fit [7]. However, despite their widespread use, ongoing research, and clinical success, research on how SSCs affect masticatory efficiency in children is limited. While existing studies have explored the impact of various malocclusions on masticatory performance [1, 9, 10], no research has specifically evaluated the effect of SSCs on masticatory efficiency. Thus, the present study aims to address this gap by assessing the impact of SSC placement on masticatory efficiency in children using two different colored chewing gums. The null hypothesis stated that there would be no alteration in the masticatory efficiency of children with and without stainless steel crown restorations.

## Methods

This pilot cross-sectional split-mouth study was conducted with the approval of the Institutional Ethics Committee (protocol reference number: 18157). The procedures followed were in accordance with the Helsinki Declaration of 1975, as revised in 2000.

### Study setting

The study population included patients receiving dental treatment at the Department of Pediatric and Preventive Dentistry, Manipal College of Dental Sciences, Mangalore. The data collection was carried out for six months between December 2021 and April 2022.

### Sample size calculation

Given that no similar studies had been published previously, the research was then conducted as a pilot study. The sample size for this research was determined on the basis of a study by Toro et al. [10], which evaluated the effect of malocclusion on masticatory performance. At a confidence interval of 95% with an anticipated standard deviation of 0.78 units, as described in the study by Toro et al. [10], with an acceptable margin of error in estimating a true population mean of 0.4, the sample size obtained was 14.61. Thus, fifteen dental patients participated in this study.

### **Study** participants

The participants were children aged 6–11 years who were in good health and accompanied by a parent or guardian capable of understanding and responding to study-related questions. Specifically, the inclusion criterion was that these children had stainless-steel crowns placed unilaterally on one or more teeth, either on the right or left side, at least one month before the study. The children received stainless-steel crowns after one of the following treatments: restoration, pulpotomy, or pulpectomy. While the type of treatment delivered before crown placement especially the vitality of the tooth could potentially confound our evaluation of the impact of SSCs on masticatory efficiency, we were unable to specify the treatments due to the challenges in obtaining a sufficient number of appropriate study participants.

Children with discernible mental or communicative disorders, those unwilling to participate, those with a crossbite, open bite, maligned teeth, and/or crowding, and those experiencing dental emergencies were excluded from the study to maintain the integrity of the data and ensure participant safety. Before enrolling participants, thorough explanations of the study procedures were provided to both the parents or guardians and the children themselves. Written informed consent was obtained from the parents or guardian, affirming their understanding of the study’s aims, procedures, and voluntary participation.

### Study procedure and data collection

Two flavours of the same brand (Trident, Perfetti Van Melle, USA, Inc.) of chewing gum were used. The flavoured cinnamon was red, and the flavoured mint was green. This brand was chosen because it provides the correct dimension, color, and texture required for the study. Half a strip of red and green chewing gum placed one on top of the other was positioned between the maxillary and mandibular teeth on the side of the mouth with no stainless-steel crowns. The child was then asked to chew this piece of chewing gum using only the teeth on that specific side for fifteen counts (the child was instructed to chew once for each count). After the chewed gum was collected, another set of chewing gum was given to the child to chew for 20 counts using the same side of the mouth. The child was asked to repeat the same chewing exercise using the teeth on the other side (where one or more teeth had well-seated stainless-steel crowns).

**Fig. 1:**
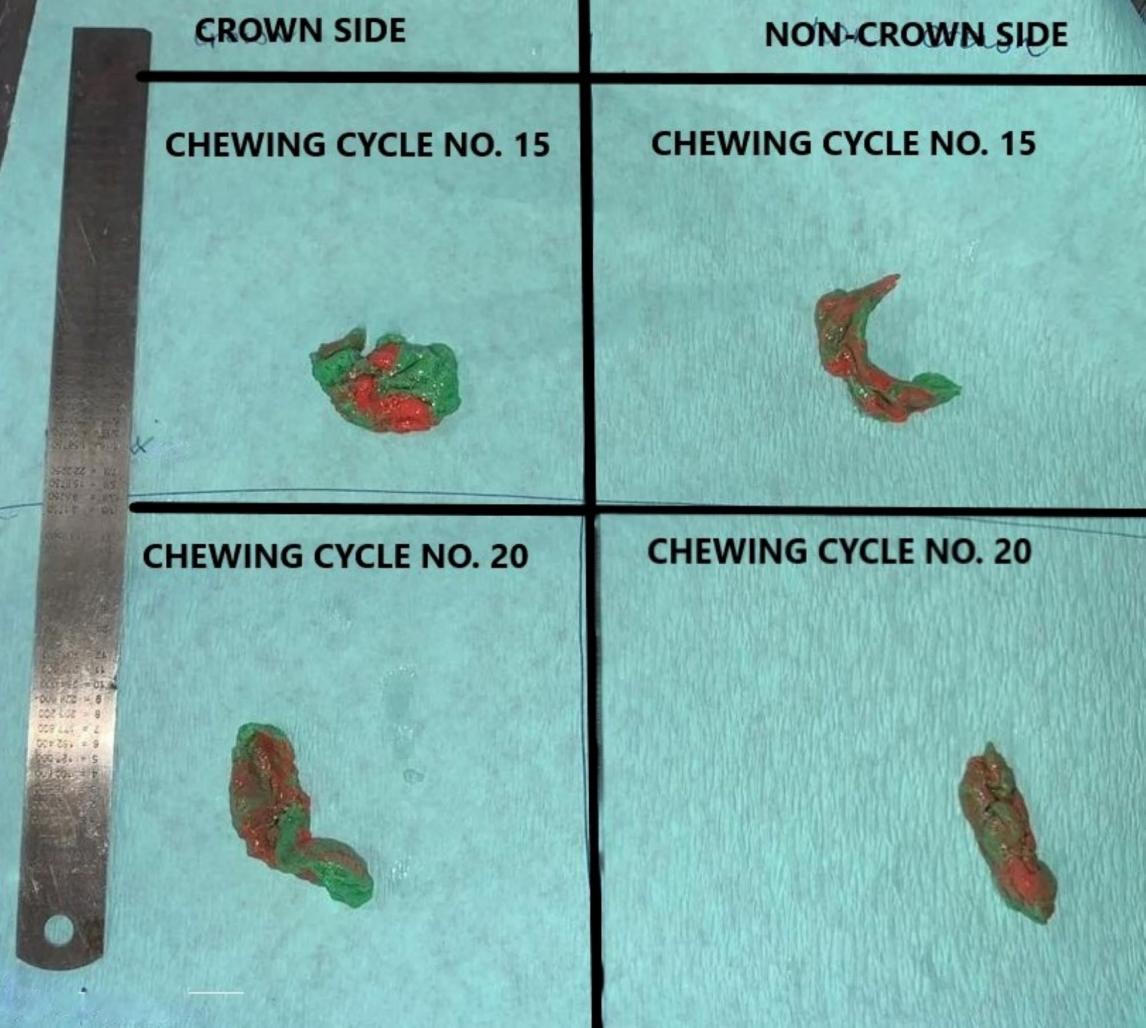
Chewed gums collected from patients following 15 and 20 chewing cycles

**Fig. 2:**
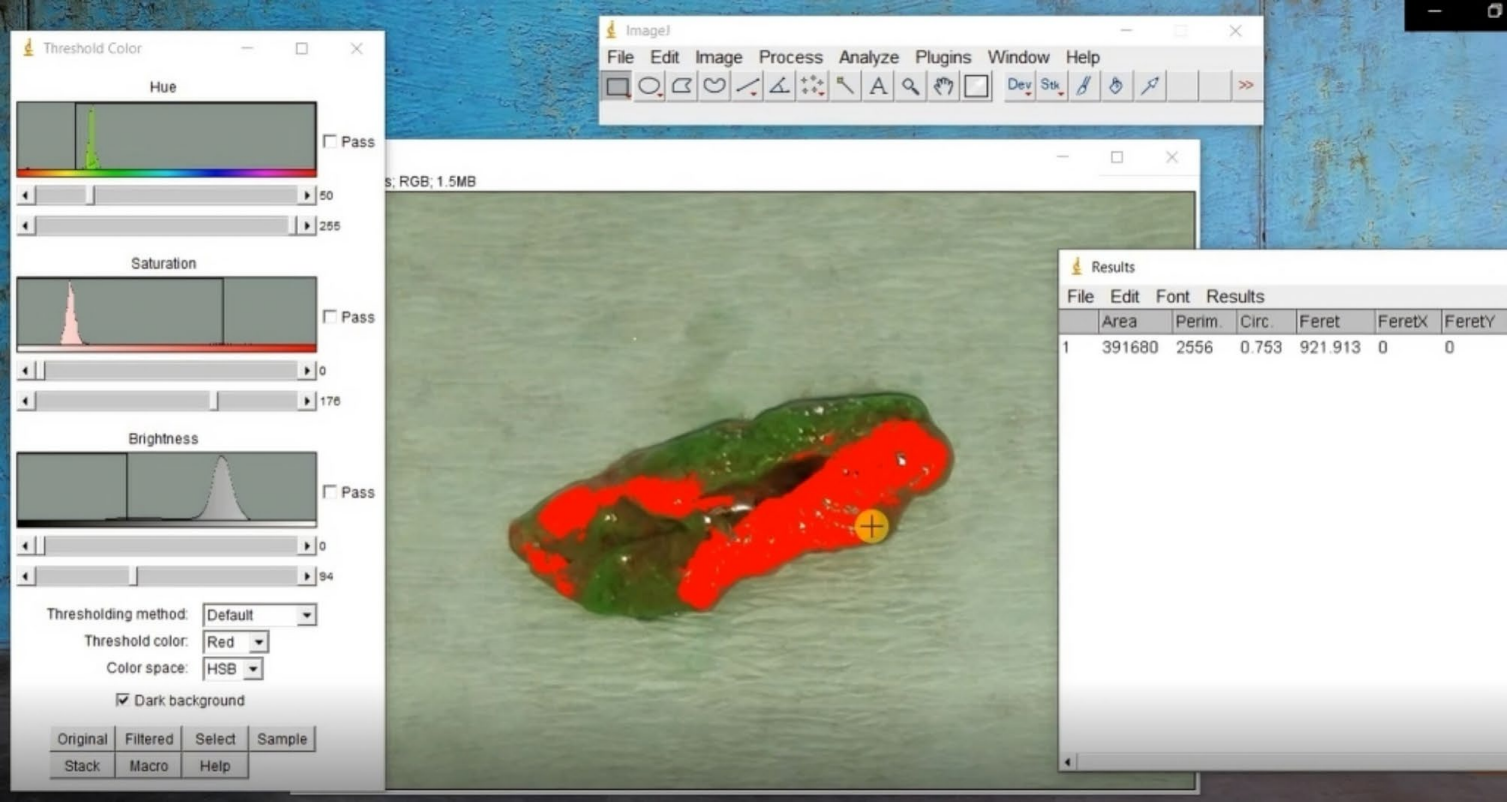
Calculation of color percentages via ImageJ software

Following every chewing cycle, the chewed gum sample was collected and photographed with a metallic measuring scale on a white background (Fig. 1). Each image was analysed via ImageJ software (Fig. 2). The masticatory efficiency was calculated as follows:

Masticatory efficiency = 100 – (Red area + Green area)/Total area) × 100.

### Blinding

The outcome assessor who carried out the ImageJ analysis and the statistician who carried out the data analysis were blinded to the study groups.

### Statistical **analysis**

The data obtained for chewing efficiency in 15 cycles and 20 cycles were compared between the crown side and the noncrown side. The Shapiro‒Wilk test ensured that the data were normally distributed. Therefore, masticatory efficiency was compared between the crown side and the noncrown side via paired t tests. All the data were processed via the SPSS (version 20.0) (SPSS, Chicago, IL, USA) software package. The significance level was set at 5% (*P* < 0.05).

As the study was cross-sectional, no missing data were encountered, ensuring that all the required information from the participants was complete and reliable for analysis. Additionally, when parametric tests were applied to the data, no outliers were found, indicating that the dataset was consistent without any extreme values that could distort the study’s conclusions or interpretations.

## Results

Among the 15 included patients, stainless steel crowns were placed on either the right side or the left side. The crowns were seated on the first permanent molars (2 patients), first primary molars (5 patients), and second primary molars (8 patients). Eight patients had crowns placed on the right side, while 7 of them had SSCs on the left side.

**Fig. 3:**
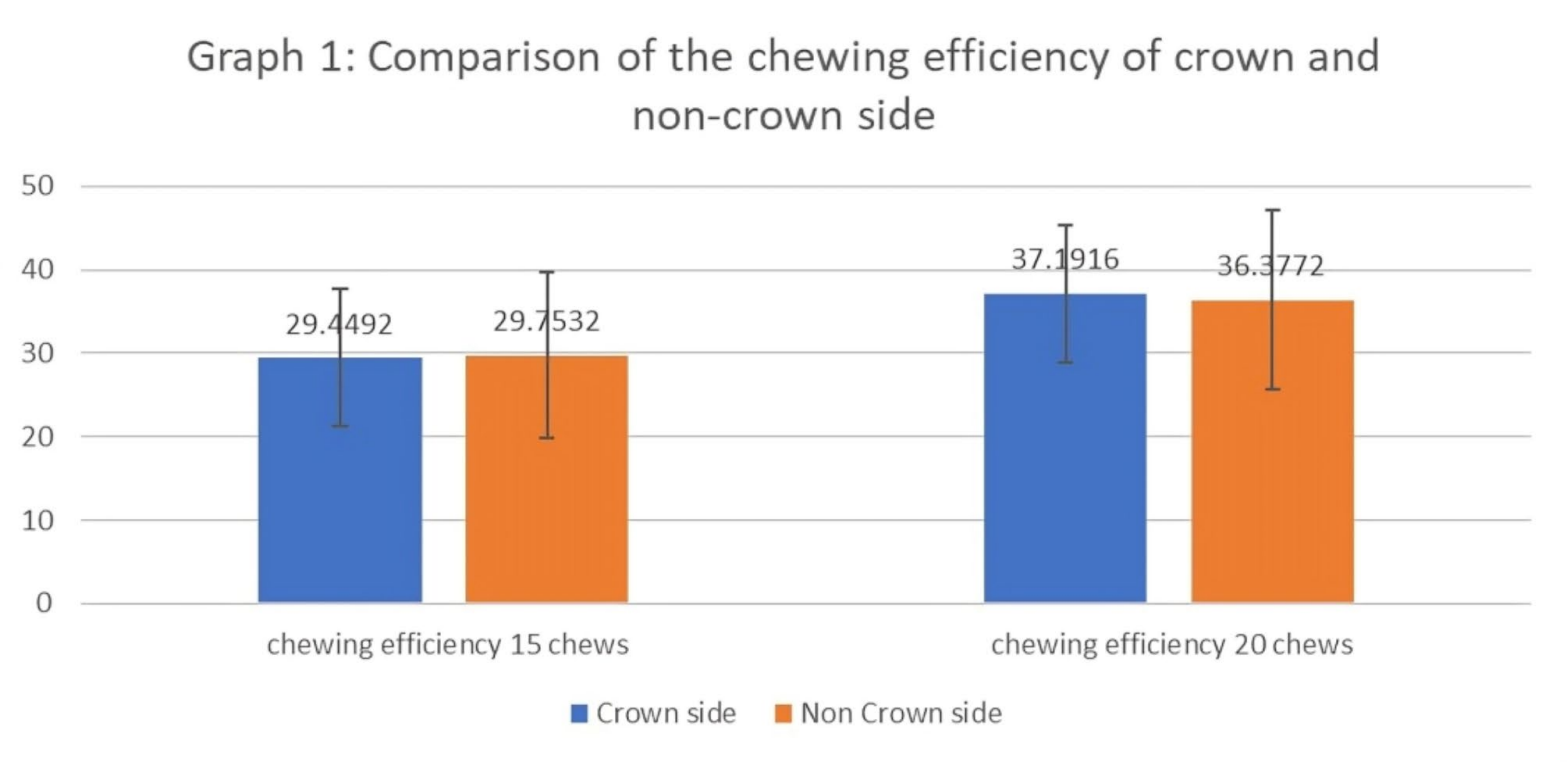
Graphical comparison of the chewing efficiency of the crown and noncrown sides

When the percentage of red and green color mixed in gum chewed on the crown side was compared with that on the noncrown side after 15 and 20 chewing cycles (Table 1), no statistically significant differences were observed (*p* = 0.869 and 0.455, respectively). Interestingly, after 15 cycles, the chewing efficiency was slightly greater on the noncrown side by 0.3, whereas after 20 cycles, it was greater on the crown side by 0.81. However, both differences were statistically nonsignificant (Table 2; Fig. 3).

**Table 1:** Comparison of the color percentages between crown side and noncrown side using paired t test

		*N*	Crown side (Mean ± SD)	Non-crown side (Mean ± SD)	Mean difference ± SD	T	*p* value
15 Times of Chewing	Green Percentage	15	40.97 ± 12.37	37.67 ± 11.95	3.3 ± 19.92	0.641	0.53
	Red Percentage	15	28.22 ± 8.73	32.39 ± 12.27	-4.17 ± 14.83	-1.09	0.29
	Mixed Percentage	15	28.9 ± 8.63	29.63 ± 10.36	-0.73 ± 16.94	-0.167	0.87
20 Times of Chewing	Green Percentage	15	39.49 ± 13.86	31.65 ± 10.61	7.84 ± 18.41	1.649	0.12
	Red Percentage	15	26.44 ± 7.69	30.47 ± 7.53	-4.03 ± 12.77	-1.222	0.24
	Mixed Percentage	15	38.16 ± 9.35	35.76 ± 11.1	2.39 ± 12.06	0.769	0.46

**Table 2:** Comparison of the chewing efficiency between crown and noncrown sides using paired t test

		*N*	Mean difference ± SD	t	*p* value
Pair 1 (15 chewing cycles)	Crown side chewing efficiency	15	-0.3 ± 16.56	-0.07	0.94
	Noncrown side chewing efficiency	15			
Pair 2 (20 chewing cycles)	Crown side chewing efficiency	15	0.81 ± 12.37	0.26	0.80
	Noncrown side chewing efficiency	15			

## Discussion

The relevance of evaluating masticatory efficiency in dentistry is well documented [11], with studies emphasizing the impact of tooth material [12–15] and cuspal angulations [16–21] on optimal chewing function. Preformed SSCs are known to cause discomfort due to premature occlusal contact immediately after placement, which typically resolves within a month [22, 23]. Gallagher et al. [8] reported that occlusal disruptions normalize within four weeks post-SSC placement. Hence, our study included children with SSCs placed at least one month prior to the study to minimize acute effects on chewing efficiency.

Among the various techniques available for measuring masticatory efficiency, methods using gum or wax are the most recent [1]. For example, Sato et al. developed a technique using a hexahedral paraffin wax cube with alternating layers of two colors and analysed masticatory efficiency through computerized image analysis of the color mix and distribution [11]. This method allows for objective evaluation of masticatory efficiency in a reproducible, precise, noninvasive, easy, and child-friendly manner [11, 24]. Schimmel et al. [25] validated this approach, demonstrating its reliability through digital image analysis via ImageJ software, which was employed in our study. They also recommended 20 chewing cycles for optimal assessment, a protocol adopted in our evaluation.

Our findings indicated greater masticatory efficiency on the noncrown side after 15 chewing cycles, suggesting quicker optimization in natural teeth. Interestingly, after 20 cycles, the efficiency on the crown side equaled and surpassed that on the noncrown side, potentially due to the durable anatomy of the crowns, with less wear and tear than tooth enamel. However, despite these observations, no statistically significant difference in masticatory efficiency was found between the crown and noncrown sides. This aligns with previous reports of initial occlusal force reduction post-SSC placement followed by recovery over time [22]. Kindelan et al. [26] documented varied opinions among children, parents, and dentists regarding masticatory efficiency post-SSC placement, underscoring the multifaceted nature of patient experiences in dental restoration studies.

Even though this study eliminated most of the confounding factors from being a split-mouth trial, it has certain limitations. This was a pilot study conducted on a limited number of samples, thus restricting the generalizability of the findings to a broader population. Additionally, not limiting to a single treatment before crown placement can also be a limitation, as the tooth being vital or nonvital may influence masticatory efficiency. Owing to the small sample size, conducting subgroup analyses to explore potential differences between different demographics or conditions was not feasible. Therefore, while the initial findings are informative, further research with larger and more diverse samples would be necessary to confirm and extend these preliminary results.

## Conclusion

Within the limitations of the present pilot study, the placement of preformed stainless-steel crowns on the deciduous molars of children did not alter masticatory efficiency.

## Data Availability

The raw data and digital images used in the study are stored locally with the corresponding author and are freely available on request.

## Declarations

### Clinical trial number

Not applicable, as it is not a randomized clinical trial.
